# Impact of 6MV photon beam attenuation by carbon fiber couch and immobilization devices in IMRT planning and dose delivery

**DOI:** 10.4103/0971-6203.26690

**Published:** 2006

**Authors:** R. K. Munjal, P. S. Negi, A. G. Babu, S. N. Sinha, A. K. Anand, T. Kataria

**Affiliations:** Rajiv Gandhi Cancer Institute and Research Centre, Rohini, Delhi, India

**Keywords:** Attenuation, base plate, carbon fiber, dosimetry, intensity modulated radiation therapy, treatment couch

## Abstract

Multiple fields in IMRT and optimization allow conformal dose to the target and reduced dose to the surroundings and the regions of interest. Thus we can escalate the dose to the target to achieve better tumor control with low morbidity. Orientation of multiple beams can be achieved by i) different gantry angles, ii) rotating patient's couch isocentrically. In doing so, one or more beam may pass through different materials like the treatment couch, immobilization cast fixation plate, head and neck rest or any other supportive device. Our observations for 6MV photon beam on PRIMUS-KXE2 with MED-TEC carbon fiber tabletop and 10 × 10 cm^2^ field size reveals that the maximum dose attenuation by the couch was of the order of 2.96% from gantry angle 120-160°. Attenuation due to cast fixation base plate of PMMA alone was of the order of 5.8-10.55% at gantry angle between 0 and 90°. Attenuation due to carbon fiber base plate alone was 3.8-7.98%. Attenuation coefficient of carbon fiber and PMMA was evaluated and was of the order of 0.082 cm^−1^ and 0.064 cm^−1^ respectively. Most of the TPS are configured for direct beam incidence attenuation correction factors only. Whereas when the beam is obliquely incident on the couch, base plate, headrest and any other immobilization device get attenuated more than the direct beam incidence. The correction factors for oblique incidence beam attenuation are not configured in most of the commercially available treatment planning systems. Therefore, such high variations in dose delivery could lead to under-dosage to the target volume for treatments requiring multiple fields in IMRT and 3D-CRT and need to be corrected for monitor unit calculations.

Multiple fields in IMRT and optimization allow conformal dose to the target and reduced dose to the surroundings and the regions of interest. Thus we can escalate the dose to the target to achieve better tumor control with low morbidity. Orientation of multiple beams can be achieved by i) different gantry angles, ii) rotating patient's couch isocentrically. In doing so, one or more beam may pass through different materials like the treatment couch, immobilization cast fixation plate, head and neck rest or any other supportive device.[1,2] Nowadays high absorption table couch tops, which required restricted gantry angles for coplanar intensity modulated radiotherapy,[3] are no more preferred in clinical use and are being replaced by low absorption carbon fiber linac table tops, which, because of their physical properties of high strength, low density, translucence, practically no sagging at the end with weight, are more favorable for radiation treatment.[4] Studies[4,5] also concluded that the attenuation of high energy photon beams by carbon fiber inserts was insignificant as compared to hardboard, copolyester and PMMA. Therefore, varieties of such carbon fiber couches and inserts are now being used in clinical practice for 3D-CRT and IMRT treatments. Original equipment manufacturers (OEM) of linacs may supply their own standard carbon fiber couch tops having different shapes and combinations of carbon fiber and foam core.[2,6–8] Attenuation of the beam through carbon fiber couch has been reported.[2,4,6,7] These studies were concerned with direct incidence of beam on the couch. Studies[1] on Sinmed BV Posisert carbon fiber support for 6MV beam have reported 8.7% beam attenuation at gantry angle of 70°. EPID-based studies[9] have also been reported on Exact treatment couch insert of carbon fiber with head and neck immobilization devices and have concluded an attenuation of 15% in clinical practice[7] has reported gantry angle dependent attenuation measurements for a standard carbon fiber couch of a linac (OEM) compared with SINMED couch inserts of the order of 4.5 and 4.2% for both left and right posterior oblique beam respectively at isocenter without using any immobilization devices. Such high absorption of beam could be attributed to attenuation by the couch and immobilization devices. So if the beam passes through these materials before entering the patients, it can cause unacceptable shift in the dose distribution, which goes unnoticed in the treatment planning systems, as most of the commercially available treatment planning systems do not account for the absorption of beam through these materials for oblique incidence of beam. We, at our center, have made an effort to evaluate the attenuation of 6MV beam on MED-TEC (USA) carbon fiber indexed (IPPS) couch and inserts, as no literature is available for this type of table top, as well as for head rest, PMMA cast fixation plate and carbon fiber plate used for patient's immobilization and support.

## Materials and Methods

**Figure 4:**
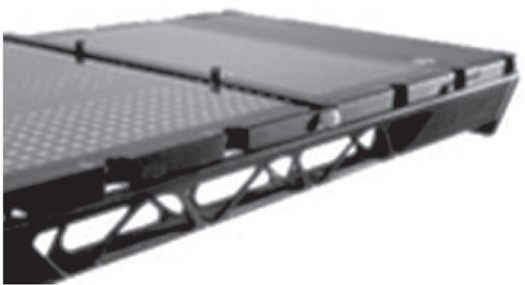
MED-TECH IPPS COUCH

We, at our center, have installed Siemens PRIMUS KXE2 having 6MV photon beam with MED-TEC (USA) carbon fiber patients couch with a tennis racket covered with Mylar sheet to reduce the beam attenuation through the couch and side rails of the couch are of cross-cross configuration [Figure 4]. We have observed, over a period, that there is practically no sagging of couch with weight even at the end of the couch.

**Figure 1:**
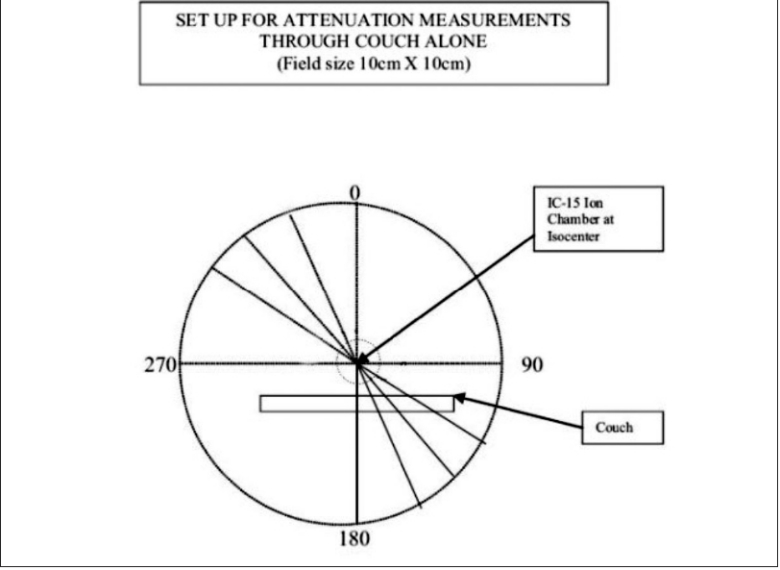
In air measurement

**Figure 2:**
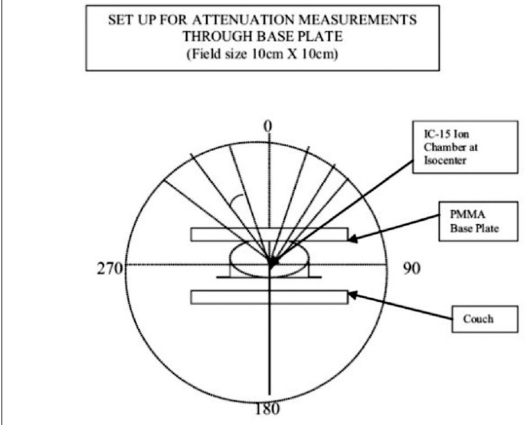
Measurement in wellhofer IMRT phantom

**Figure 3:**
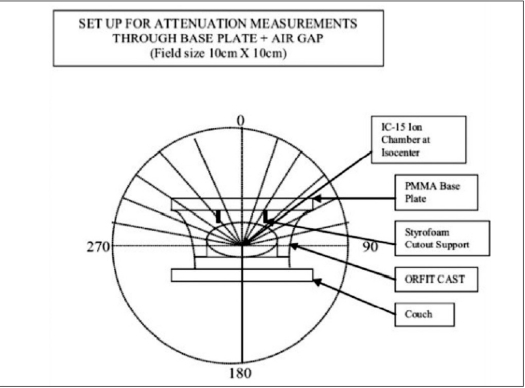
Measurement in wellhofer IMRT phantom

As a part of QA program, CT of Wellhofer IMRT phantom was done and planned on TPS for IMRT with multiple fields. Experimental dosimetric studies were done to evaluate the dose planned and measurement on the machine in the phantom. It was observed that the measured dose at some of the gantry angles was different than the TPS plan. This variation in dose evaluation initiated us to investigate the problem in depth. Therefore, independent dosimetric studies were carried out to evaluate attenuation due to couch [Figure 1], with cast fixation plate in close contact with phantom [Figure 2] and with air gap between base plate and phantom along with orfit cast [Figure 3] to simulate head and neck geometry with Silverman's headrest. Dose measurements were carried out with IC-15 ion chamber with CAPINTEC-CII Wellhofer electrometer. PMMA base plate was placed on the phantom supported on a Styrofoam cutout to create air gap between phantom body and base plate to simulate head and neck IMRT treatment using Silvermans headrest. Orfit cast was made. The idea of making cast was to do CT with cast to evaluate the dose verification in IMRT Treatment planning and dose delivery with contour taking into account the condition with cast and without cast [Figures 5A and 5B]. We have also evaluated the density and measured the absorption coefficient of carbon fiber sheets supplied by M/s POCL, and PMMA sheets supplied by M/s Midas India used under study.

**Figure 5a:**
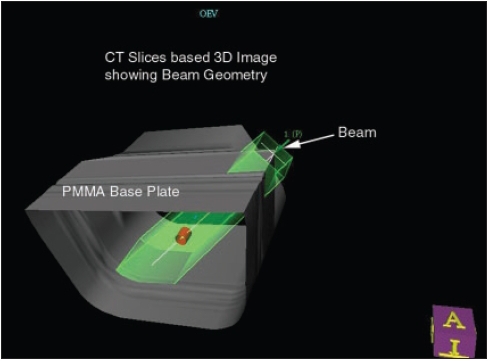
CT based 3D image with beam projection

**Figure 5b:**
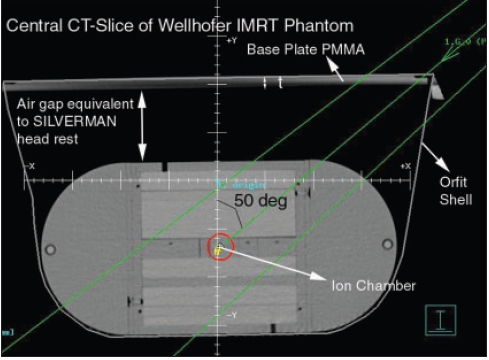
Central ct slice of wellhofer IMRT phantom with ion chamber

### Observations

**Graph 1:**
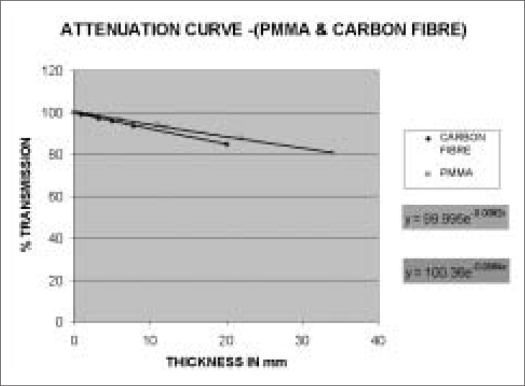
Attenuation curve (PMMA and carbon fiber)

The following observations have been recorded:

1. The detector with build-up cap was placed at isocenter on a stand in air above the tennis racket to evaluate attenuation due to couch alone. Readings were taken at gantry angles of 10-degree intervals from 0-360°. The maximum dose attenuation observed was of the order of 2.96% from gantry angles 120-160° and 240-200° [Table 1].
2. Attenuation due to cast fixation base plate of PMMA (12 mm thick) alone was of the order of 4.85-10.55% at gantry angles between 0 and 90°, with maximum value at gantry angle of 60°. In this case, the base plate was placed above the Wellhofer IMRT phantom to avoid attenuation due to couch for measurements and detector was placed in the phantom at isocenter [Table 3].
3. Attenuation due to carbon fiber plate (8 mm thick) alone was also studied by placing over the IMRT phantom and measurements were done from 0-90° gantry angle with an interval of 10 degrees Maximum beam attenuation of 7.98% was observed at gantry angle of 60° [Table 3].
4. Measurements were also done to study the effect of air gap between PMMA base plate and phantom, as in the case of head and neck treatment using Silverman's head rest for support. In this study, we have placed the base plate above the phantom with Styrofoam cut-out support, and orfit cast was made. Dose variation was observed from 5.82-7.42% from gantry angles between 0 and 90° [Table 2].
5. Attenuation coefficients (μ), measured for carbon fiber sheet and PMMA used for base plate, were of the order of 0.082 cm^−1^ and 0.064 cm^−1^ respectively for small field size of 2 × 2 cm (Graph 1). Thus for normal incidence, carbon fiber thicknesses of 1, 2, 3, 4 mm will attenuate 2 × 2cm 6MV beam by 0.820, 1.626, 2.430 and 3.23% respectively.
6. Measured density of carbon fiber and PMMA used was of the order of 1.39 gm/cc and 1.18 gm/cc respectively.
7. The purpose of measuring attenuation coefficient was to evaluate and correlate the thickness (t) of carbon fiber or PMMA base plate used for immobilization purposes and of the couch for normal or oblique incidence of beam traversed through these materials for oblique angle (F). Transmission of beam was calculated by the equation angle (φ). Transmission of beam was calculated by the equation–${\text{I}}_{\text{t}}{\text{=I}}_{\text{0}}{\text{e}}^{\text{-μt/CosΦ}}$
  Two sets of studies were carried out independently to verify-
  - Contour was outlined for phantom alone: MUs were calculated for a dose of 100cGy at isocenter in the PLATO-SUNRISE TPS at different gantry angles for a field size of 10 × 10 cm and the doses were measured in the phantom at isocenter with IC-15 ion chamber using the calculated MUs [Table 4].
  - In another case the contour was outlined along with base plate and cast, and MUs were calculated for a dose of 100 cGy at isocenter in the TPS at various gantry angles. The doses were measured in the phantom using the TPS-calculated MUs [Table 4].

**Table 1:** Beam attenuation due to couch alone [Figure 1]

Gantry angle	% Attenuation due to couch alone (degree)
180	1.22
160	1.40
140	1.92
120	2.965
100	0.01
90	0.00

**Table 2:** Beam attenuation due to PMMA base plate (12 mm) + air gap simulating head and neck RT geometry [Figures 2, 3]

*Gantry angle*	*Path length (mm) PMMA*	*PMMA base plate + air gap % attenuation (degree)*
0	12.00	5.82
10	12.185	5.84
20	12.77	6.27
30	13.86	6.97
40	15.66	7.36
50	18.67	7.42
60	0.0	1.26
70	0.0	0.00
80	0.0	0.00
90	0.0	0.00

At gantry angle 60, part of the beam is passing through the base plate

**Table 3:** Beam attenuation due to PMMA base plate (12 mm) without air gap and carbon fiber base plate

*Gantry angle (degree)*	*Path length (mm) PMMA*	*PMMA base plate on the phantom % attenuation*	*Path length (mm) Carbon fibre*	*Carbon fiber plate on the phantom % attenuation*
0	12.00	4.85	8.0	3.68
10	12.185	5.19	8.12	3.70
20	12.77	5.28	8.51	3.93
30	13.856	5.78	9.23	4.23
40	15.66	6.28	10.44	5.00
50	18.67	7.81	12.44	6.22
60	24.00	10.55	16.0	7.98
70	35.00	10.31	0.0	0.00
80	0.0	0.00	0.0	0.00
90	0.0	0.00	0.0	0.00

It is to mention that half of the beam is passing through the PMMA base plate at gantry angle 70 degree

**Table 4:** TPS-calculated MUs for 100cGy dose at isocenter in phantom *vs.* measured dose

*Gantry Angle*	*TPS Cale. MU contour with base late*	*Measured dose cGy*	*TPS Cale. MU Contour Phantom alone*	*Measured dose cGy*	*% diff in dose delivery*
0	123	98.406	119	95.13	3.33
10	124	98.50	119	94.31	4.25
20	126	98.859	120	93.22	5.70
30	133	98.95	125	92.95	6.06
40	139	98.45	132	92.41	6.13
50	154	98.72	143	90.77	8.05
60	154	98.81	154	98.95	−0.14
70	161	98.99	161	98.86	0.13

## Conclusion

From the above observations, it has been concluded that there is a significant attenuation of beam through couch, inserts and immobilization devices. Attenuation of beam is a function of angle of beam incidence based on normal incident beam measurements. Experimental data should be acquired on individual bases and should be used to account for the beam attenuation in monitor unit calculations in the TPS for clinical practice. However, these correction factors should be applied with caution, with other aspects of IMRT treatment planning and dose delivery, as the calculated dose is likely to increase for the patient where beam delivered remains un-attenuated through these materials.

It has been observed that when the phantom is contoured along with cast and base plate, the TPS-calculated and measured doses are within 1.5%; whereas if phantom is contoured without base plate, the calculated dose and measured/delivered dose is as low as by 8.1% at gantry angle 50°, where the radiation path length in PMMA is maximum, i.e., 18.67 mm in PMMA base plate [Table 4]. Therefore, it is suggested that if the body contours are drawn along with base plate and supportive devices, the dose delivery is within 1.5%. The TPS has been configured for different materials' densities and other parameters for attenuation through which the beam traverses, like the base plate, headrest or any other supportive devices. The maximum beam attenuation through couch alone in our study is 2.965%, which we are not able to take into account as our CT table, though it has a flat top, is made of material other than carbon fiber, whereas treatment couch is MED-TEC carbon fiber; so in CT slices, we cannot contour along the couch. Thus, couch attenuation correction factor needs to be taken into account manually as per the data generated experimentally. If CT table and treatment couch both are of same material, then couch should also be included along with body contour to account for beam attenuation in the TPS calculations.
